# Effect of Nursing Managers’ Ethical Leadership on Clinical Nurse Empowerment, Performance, and Organizational Commitment

**DOI:** 10.1097/jnr.0000000000000689

**Published:** 2025-08-01

**Authors:** Jihun Kim, Seok Hee Jeong, Hee Sun Kim, Myung Ha Lee

**Affiliations:** 1 Department of Nursing, Jeonbuk National University Hospital, Jeonju, Republic of Korea; 2 Research Institute of Nursing Science, College of Nursing, Jeonbuk National University, Jeonju, Republic of Korea; 3 College of Nursing, Jeonbuk National University, Jeonju, Republic of Korea

**Keywords:** leadership, empowerment, work performance, nurses, mediation analysis

## Abstract

**Background:**

Effective leadership by nursing unit managers can empower nurses, which is known to positively affect their performance and organizational commitment. However, a paucity of studies has investigated the mediating role of empowerment on the relationship between ethical leadership and the performance and organizational commitment of nurses.

**Purpose:**

This study was designed to investigate empowerment as a mediator between the ethical leadership of nursing managers and the professional performance and organizational commitment of clinical nurses.

**Methods:**

Two hundred and twenty nurses working in tertiary general hospitals in South Korea participated in an online survey conducted from August 1 to September 30, 2020. Mediation analysis was performed using the Hayes PROCESS macro for SPSS.

**Results:**

Empowerment was found to mediate the relationship between ethical nursing leadership and, respectively, clinical nurse performance and organizational commitment. Notably, ethical nursing leadership was also found to directly influence organizational commitment in clinical nurses.

**Conclusions/Implications for Practice:**

Empowerment significantly and positively mediates the influence of ethical nursing leadership on key nursing productivity outcomes such as nursing performance and organizational commitment. Thus, empowering clinical nurses is critical to improving their performance and organizational commitment. Ethical leadership by nursing unit managers can help empower nurses and improve their performance.

## Introduction

In today’s increasingly competitive and dynamic health care environments, medical institutions often adopt entrepreneurial management strategies to increase organizational productivity. Clinical nurses account for the largest proportion of human resources in health care settings. Moreover, their frequent and regular interactions with patients give clinical nurses an outsized influence on organizational profitability and performance.

Failure to provide the best possible nursing performance can have harmful consequences for patients (Rapin et al., 2019). Nursing performance reflects a nurse’s ability to provide nursing care that effectively improves a patient’s condition (Lee & Ko, 2010; Rapin et al., 2019). As a main indicator of nursing productivity, nursing performance relates directly to organizational performance (Lee & Ko, 2010). Organizational commitment is expected of staff across all organizations, including medical institutions. Organizational commitment refers to the extent to which an individual self-identifies, engages with, and supports their employer (Gholami et al., 2019). Organizational commitment is a factor of influence on organizational effectiveness, and continuous efforts have been made in the field of management to enhance staff organizational commitment. Previous studies have identified nursing unit manager leadership as a key determinant of professional performance and organizational commitment in clinical nurses (Kim et al., 2022).

Clinical nurses work closely with nursing unit managers, who are typically their immediate supervisors, and the leadership displayed by these managers strongly influences the work of clinical nurses. The positive influence of various types of leadership on clinical nurses has been demonstrated empirically in several studies in the field of nursing management (Bedi et al., 2016; Kim et al., 2022). Effective leadership by managers in clinical settings helps reduce adverse patient outcomes and promotes positive staff development (Labrague et al., 2021; Lee et al., 2023).

Performance has been the traditional focus of leadership within organizations. However, after the Enron scandal in the United States, characterized by the unethical actions of its leaders, the focus of leadership has shifted toward leadership ethics, i.e., ethical leadership (Ayan & Baykal, 2023; Johns, 2024). This scandal highlighted the importance of ethical leadership, demonstrating how unethical practices can eclipse performance and impact global socioeconomic landscapes, and underscored the essentiality of ethical conduct in leadership. Organizational science research has shown that ethical leadership promotes trust and transparency in organizations (Brown & Treviño, 2006; Kalshoven et al., 2011). Ethical leaders build trust among organizational members through honesty, fairness, and consistency, and strengthen organizational culture through transparent communication (Brown & Treviño, 2006). In addition, ethical leadership is important in setting the moral standards and values for an organization, with ethical leaders raising the standard for ethical behavior throughout their organizations by leading by example and practicing ethical values (Zhang et al., 2018). Recent meta-analyses conducted by Bedi et al. (2016), Kim et al. (2022), and others have substantiated the significant role of ethical leadership in organizations, showing systematically how ethical leadership contributes to a range of positive outcomes, including enhanced job satisfaction, stronger organizational citizenship behaviors, and a more ethical climate. This body of research delineates the mechanisms by which ethical leadership fosters individual and organizational performance through the encouragement of ethical conduct, enhancement of job engagement, and amelioration of negative factors such as work-related stress and turnover intention. Despite these insights, a notable gap in the literature remains regarding the impact of ethical leadership on nursing performance and organizational commitment. Given the critical role of nurses in health care and the unique challenges they face, exploring the implications of ethical leadership within the nursing context is imperative. Thus, the objective of this study was to elucidate how ethical leadership can concretely benefit nursing performance and strengthen organizational commitment among nurses, thereby contributing to the overall efficacy and ethical standards of health care institutions.

Empowering staff is an important role of managers (Marquis & Huston, 2006), as empowered staff tend to feel more confident in their ability to perform important tasks for their organization (Fragkos et al., 2020). A recent meta-path analysis revealed that empowerment can mediate the effect of transformative nursing unit leadership on the performance and job satisfaction of clinical nurses (Kim & Jeong, 2020). Empowerment has a mediating effect on the influence of authentic or servant leadership on clinical nurse organizational commitment (Choi & Ahn, 2016; Lee et al., 2015). Also, the findings of recent studies support a positive correlation between empowerment and both the servant leadership (Lee et al., 2015) and transformational leadership (Kim & Jeong, 2020) of nursing unit managers. Furthermore, studies have consistently demonstrated that empowerment positively influences both nursing performance (Song et al., 2023; Wong & Laschinger, 2013) and organizational commitment among clinical nurses (Gholami et al., 2019). Despite these substantial findings, a significant gap in scholarly knowledge still exists with regard to the mediating role of empowerment in the nexus between ethical leadership in nursing managers and the professional performance and organizational commitment of clinical nurses. To address this gap, ethical leadership in nursing managers was posited in this study as exerting an indirect effect on nursing performance and organizational commitment through the pathway of empowerment. Specifically, the influence of ethical leadership on clinical nurse performance and organizational commitment was hypothesized to be substantially mediated by empowerment.

The aim of this study was to determine the importance of empowerment as a mediator in the relationship between ethical leadership in nursing managers and the professional performance and organizational commitment of clinical nurses.

## Methods

### Study Design and Participants

This was a nationwide, online cross-sectional survey study of clinical nurses working in tertiary general hospitals. The inclusion criteria were (a) clinical nurses currently working at a tertiary general hospital with working experience of ≥1 year and (b) working as part of a nursing unit under the supervision of a nursing unit manager. The rationale for selecting nurses with at least 1 year of experience was that it takes a minimum of 8 to 12 months for a nurse to adapt to new roles and tasks (Duchscher, 2008). The G* Power program 3.1.9.2 was used to calculate the required sample size. Based on a significance level (α) of .05, a power (1-β) of 0.90, a medium effect size of *d* = 0.5, and 13 predictors, the estimated sample size was 162. A medium effect size was selected because a meta-analysis of the effect of ethical leadership showed a medium effect size for ethical leadership and organizational commitment (0.40; Bedi et al., 2016). After factoring in an estimated nonresponse rate of 25%, a minimum of 220 participants was targeted for this study.

### Data Collection

The participants completed an online survey, including self-reported questionnaires, from August 1 to September 30, 2020. Recruitment posters were uploaded to a popular social network and an online community for nursing professionals. Snowball sampling was used to facilitate enrollment. Interested nurses accessed the online survey through either a QR code or a link to the questionnaire. A total of 220 questionnaires were collected and used in the final data analysis.

### Data Collection Tools

#### General Characteristics

General characteristics consisted of sociodemographic and work-related characteristics, including age, gender, marital status, region, educational level, total length of clinical career, duration working in current unit, position at work, work type, current work unit, and nursing management fee. In the field of health care research, accurately quantifying the staffing levels and workload of nurses, while critical, is often challenging. Thus, to overcome this issue, the nursing management fee was adopted in this study as a surrogate measure of workload. This fee, an additional hospitalization surcharge, reflects the ratio of nursing staff to inpatients, thus making this fee a reliable proxy for evaluating nursing care intensity and workload in South Korea.

#### Ethical Leadership

Ethical leadership was assessed using the K-ELW (Kim & Park, 2015), which is the Korean version of the Ethical Leadership at Work (ELW) questionnaire developed by Kalshoven et al. (2011). ELW and K-ELW were validated at the time of their development using factor analysis to ensure validity (Kalshoven et al., 2011; Kim & Park, 2015). The K-ELW contains 31 questions assessing 7 factors (people orientation, fairness, power sharing, concern for sustainability, ethical guidance, role clarification, and integrity). Example items include “Explains what is expected from employees in terms of behaving with integrity” and “Ensures that employees follow codes of integrity.” Each question is rated on a 5-point Likert scale ranging from 1 (*strongly disagree*) to 5 (*strongly agree*). This measure of ethical leadership has been widely utilized in a range of countries, supporting its wide-ranging applicability and relevance in the field of nursing. Moreover, its use in recent studies in China and Korea (Kim et al., 2022; She et al., 2023) exemplifies its continuing relevance and validity across diverse cultural contexts, supporting its robustness and suitability for the current research purpose. Responses to negative questions were reverse-coded. Higher scores indicate higher levels of participant self-rated ethical leadership in nursing managers. The Cronbach’s α for this tool was .90 in Kalshoven et al. (2011), .94 in Kim and Park (2015), and .95 in this study.

#### Empowerment

Empowerment was measured using the Texts of Items Measuring Empowerment, a tool developed by Spreitzer (1995) and modified for clinical nurses by Kim and Lee (2001). The instrument was validated using factor analysis at the time of its development (Spreitzer, 1995). This questionnaire contains 12 questions assessing 4 factors (meaning, competence, self-determination, and impact). Each question is scored on a 5-point Likert scale, ranging from 1 (*strongly disagree*) to 5 (*strongly agree*), with higher scores indicating higher levels of clinical nurse empowerment. The modified instrument used in this study was investigated within the nursing profession in Canada (Harbridge et al., 2023), with the results supporting its validity and relevance in health care settings. Example items include “I am self-assured about my capabilities to perform my work activities” and “I have significant autonomy in determining how I do my job.” The Cronbach’s α for this questionnaire was .72 in Spreitzer (1995), .89 in Kim and Lee (2001), and .89 in the current study.

#### Nursing Performance

The nursing performance measurement tool used in this study was originally developed by South Korean researchers (Ko et al., 2007) and is widely used in South Korea (Kim & Yi, 2023). The instrument was validated at the time of its development using factor analysis (Ko et al., 2007), and its frequent use in research highlights its cultural relevance and effectiveness in assessing nursing performance. This tool has 17 questions addressing 4 factors (performance capability, performance improvement, performance attitude, and nursing process application). Each question is rated on a 5-point Likert scale ranging from 1 (*strongly disagree*) to 5 (*strongly agree*), with higher scores indicating better nursing performance. Example items include “I understand prescriptions well and plan within the given time frame to perform tasks accurately and without errors or omissions” and “I perform my nursing duties accurately and completely.” The Cronbach’s α for this questionnaire was .92 in Ko et al. (2007) and .89 in this study.

#### Organizational Commitment

Organizational commitment was measured using the Organizational Commitment Questionnaire (OCQ) developed by Mowday et al. (1979) and translated into Korean by Lee (1998). OCQ was validated at the time of its development using factor analysis (Mowday et al., 1979). This tool consists of 15 questions scored on a 7-point Likert scale ranging from 1 (*strongly disagree*) to 7 (*strongly agree*), with higher total scores indicating greater organizational commitment. Example items include “I think my hospital is one of the best places I could work for” and “I make more effort than others to improve my organization.” Its consistent reliability across different studies and time frames and prior application to nursing contexts (Cho et al., 2021) underscores the tool’s robust validity and relevance to this research. Such ongoing validation efforts affirm the suitability of using the OCQ to assess current organizational commitment among nurses. The Cronbach’s α of the OCQ was .94 in Mowday et al. (1979), .91 in Lee (1998), and .92 in this study.

### Data Analysis

Data analyses were performed using SPSS Statistics 23.0 (IBM Corp., Armonk, NY, USA). Descriptive statistics and constant variance tests were performed. For homogeneous data, the independent *t* test and one-way analysis of variance were performed with a post hoc Scheffé test. For heterogeneous data, the Welch test was performed as a two-way variance test. Pearson’s correlation coefficients were calculated. Prior to conducting a regression analysis, multicollinearity (*r*<.80), Durbin-Watson index (d_U_ <d<4-d_U_), tolerance (>0.1), and variance inflation factor (VIF <10) were tested and accepted. The hypothesized model showing the influence of ethical leadership (the independent variable) on nursing performance (first dependent variable) and organizational commitment (second dependent variable) via empowerment (the mediating variable) was tested. The mediation role was tested using multiple regression analysis and a simple mediation model applying the PROCESS macro Model 4. The statistical significance of the mediating effect was tested using bootstrapping with resampling (10,000 iterations) and a 95% bias-corrected bootstrap confidence interval (CI). Cronbach’s α was calculated to determine the internal consistency of each measure.

### Ethical Considerations

This study was approved by the institutional review board of Jeonbuk National University (IRB NO: 2020-07-013-001). The main ethical considerations involved participant consent, privacy, and confidentiality. The consent form informed participants regarding their right to refuse participation and to drop out at any stage of the study without any negative consequences.

## Results

### General Characteristics

In terms of participant characteristics (*N* = 220), the average age was 28.40 (*SD* = 4.76) years, 194 (88.2%) were female, 175 (79.5%) were single, and 68 (30.9%) self-identified as religious. Most (*n* = 201, 91.4%) held a 3-year nursing college or a 4-year bachelor’s degree. Their mean clinical career was 5.07 (*SD* = 4.63) years, and the mean duration working in the current unit was 3.11 (*SD* = 2.77) years. The most common working types reported were staff (*n* = 199, 90.5%) and shift (*n* = 206, 93.6%), and the most common working unit reported was the medical/surgical ward (*n* = 128, 58.2%). A slight majority (58.6%, *n* = 129) worked in hospitals, corresponding to a grade 1 nursing management fee (Table 1).

**Table 1:** Differences in Nursing Performance and Organizational Commitment, by General Characteristics (*N* = 220)

Variable	*n* (%)	Nursing Performance				Organizational Commitment			
		Mean	*SD*	*t* / *F*	*p*	Mean	*SD*	*t* / *F*	*p/*Scheffé test
Age (years; mean and *SD*)	28.40 (4.76)			1.51	.223			1.44	.240
23–29	154 (70.0)	3.69	0.47			3.90	0.95		
30–39	61 (27.7)	3.81	0.47			3.83	1.10		
≥40	5 (2.3)	3.80	0.62			4.61	1.15		
Gender				2.33	.021			−1.91	.064
Female	194 (88.2)	3.76	0.45			3.86	1.02		
Male	26 (11.8)	3.53	0.56			4.18	0.77		
Marital status				0.57	.571			0.09	.930
Single	175 (79.5)	3.74	0.47			3.90	1.01		
Married	45 (20.5)	3.69	0.46			3.89	0.97		
Religion				−0.43	.669			−0.82	.413
None	152 (69.1)	3.72	0.48			3.86	0.94		
Yes	68 (30.9)	3.75	0.44			3.98	1.13		
Educational level				−1.48	.139			−0.66	.511
3- and 4-year bachelor	201 (91.4)	3.72	0.48			3.88	1.00		
≥Master degree	19 (8.6)	3.88	0.38			4.04	1.01		
Total length of clinical career (years; mean and *SD*)	5.07 (4.63)			1.42	.237			1.25	.292
1–2.9	106 (48.2)	3.68	0.48			4.00	1.02		
3–4.9	37 (16.8)	3.85	0.43			3.92	0.95		
5–9.9	52 (23.6)	3.77	0.48			3.67	1.01		
≥10	25 (11.4)	3.70	0.46			3.92	0.95		
Total time in current work unit (years; mean and *SD*)	3.11 (2.77)			2.44 ^a^	.094			3.67	.027 ②>③
① 1–2.9	150 (68.2)	3.71	0.49			3.96	1.00		
② 3–4.9	32 (14.5)	3.87	0.36			4.06	0.95		
③ ≥5	38 (17.3)	3.69	0.45			3.51	0.96		
Position at work				−2.10	.037			0.52	.606
Staff nurse	199 (90.5)	3.71	0.47			3.91	0.96		
Charge nurse	21 (9.5)	3.93	0.46			3.76	1.34		
Work type				0.84	.402			1.84	.067
Nonshift regular	14 (6.4)	3.83	0.58			4.37	0.95		
2 or 3 shifts	206 (93.6)	3.72	0.46			3.87	1.00		
Current working unit				0.86	.425			1.69	.188
Medical or surgical	128 (58.2)	3.76	0.45			3.79	1.01		
ICU	52 (23.6)	3.70	0.49			4.01	1.02		
OR, AN	40 (18.2)	3.66	0.50			4.08	0.93		
Nursing management fee				2.79	.006			2.15	.033
1st grade	129 (58.6)	3.80	0.47			4.02	1.03		
2nd, 3rd grade	91 (41.4)	3.63	0.46			3.73	0.94		

*Note.* ICU = intensive care unit; OR = operating room; AN = anesthesia.

^a^ Welch test.

### Ethical Leadership, Empowerment, Nursing Performance, and Organizational Commitment

The mean participant-rated ethical leadership in nursing managers score was 3.07 ± 0.67 (out of 5); the mean score for self-rated empowerment was 3.54 ± 0.52 (out of 5); the mean score for self-rated professional performance was 3.73 ± 0.47 (out of 5); and the mean score for self-rated organizational commitment was 3.90 ± 1.00 (out of 7; Table 2).

**Table 2:** Levels of Ethical Leadership (Nursing Unit Managers) and Empowerment, Nursing Performance, and Organizational Commitment (Clinical Nurses; *N* = 220)

Variable	Possible Range	Actual Range	Mean±*SD*	Skewness	Kurtosis
Ethical leadership	1–5	1.26–4.84	3.07±0.67	−0.25	0.09
Empowerment	1–5	2.08–5.00	3.54±0.52	−0.19	0.19
Nursing performance	1–5	2.59–4.94	3.73±0.47	0.15	−0.11
Organizational Commitment	1–7	1.00–6.60	3.90±1.00	−0.15	0.22

### General Characteristic–Related Differences in Nursing Performance and Organizational Commitment

Professional performance was found to be significantly associated with gender, position at work, and nursing management fee (*p* < .05 for all). Organizational commitment was found to be significantly associated with duration working in the current unit and nursing management fee (*p* < .05 for both; Table 1).

### Correlations among Ethical Leadership, Empowerment, Nursing Performance, and Organizational Commitment

Professional performance was found to be positively correlated with ethical leadership (*r* = .26, *p* < .001) and empowerment (*r* = .69, *p* < .001), while organizational commitment was found to be positively correlated with ethical leadership (*r* = .47, *p* < .001) and empowerment (*r* = .45, *p* < .001).

### Ethical Leadership and Nursing Performance: Mediating Effect of Empowerment

**Figure 1:**
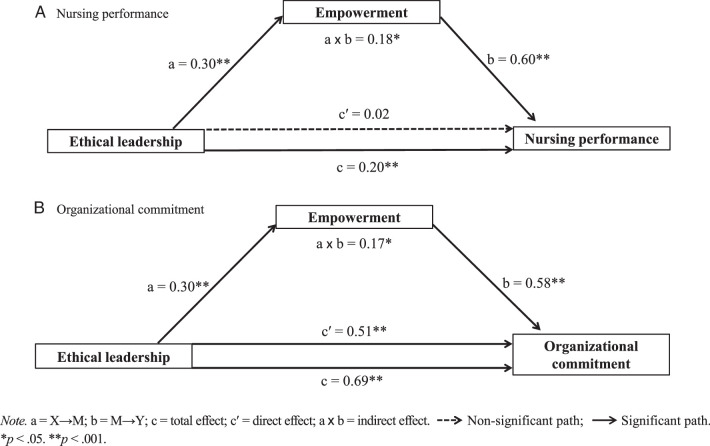
Mediating Effects of Empowerment on the Relationship Between Ethical Leadership and, Respectively, Nursing Performance and Organizational Commitment

The mediating effect of empowerment on the relationship between ethical leadership in nursing managers and professional performance in clinical nurses was analyzed. To control for exogenous variables that significantly influence nursing performance, gender, position, and nursing management fee were treated as dummy variables. As shown in Table 3 and Figure 1A, the direct path from ethical leadership to empowerment was statistically significant (X→M, *B* = 0.30, *p* < .001), while that from ethical leadership to nursing performance was not (X→Y_1_, *B* = 0.02, *p* = .630). Also, the path from empowerment to nursing performance was statistically significant (M→Y_1_, *B* = 0.60, *p* < .001). Furthermore, the mediating model centered on ethical leadership, empowerment, and nursing performance revealed a significant positive total effect (*B* = 0.20, *p* < .001). In the bootstrapping results for an indirect effect (X→M→Y_1_), the path was statistically significant (95% CI [0.113, 0.256]), indicating the relationship between ethical leadership and nursing performance is positively mediated by empowerment. Finally, ethical leadership was shown to significantly and positively influence empowerment in clinical nurses, which in turn had an improvement effect on nursing performance.

**Table 3:** Mediating Effect of Empowerment on the Relationship Between Ethical Leadership and Clinical Nurse Performance (*N* = 220)

Variable	Path	Empowerment (M)				Path	Nursing Performance (Y_1_)			
		*B*	*SE*	β	*p*		*B*	*SE*	β	*p*
Ethical leadership (X)	a	0.30	.05	0.39	<.001	c’	0.02	0.04	0.03	.630
Empowerment (M)						b	0.60	0.05	0.66	<.001

Dependent Variable	Independent Variable	Mediator Variable	Indirect Effect							
			*B*		Boot *SE*		Boot 95% CI			
Performance (Y1)	Ethical leadership	Empowerment	0.18		0.04		[0.113, 0.256]			

*Note.* Tolerance = .81–.99; VIF = 1.01–1.24; Durbin-Watson = 1.966 (du = 1.826 < d < 4-du = 2.174); *B* = unstandardized estimates; *SE* = standardized error; β = standardized estimates; CI = confidence interval; X = Independent variable; M = mediator variable; Y_1_ = first dependent variable; Covariate variables = gender (male = 0, female = 1), position (staff nurse = 0, charge nurse = 1), nursing management fee (2nd or 3rd grade = 0, 1st grade = 1).

### Ethical Leadership and Organizational Commitment: Mediating Effect of Empowerment

The mediating effect of empowerment on the relationship between ethical leadership in nursing managers and organizational commitment in clinical nurses was analyzed. Duration working in the current unit and nursing management fee, both of which significantly influenced organizational commitment, were treated as dummy variables. As shown in Table 4 and Figure 1B, the direct path from ethical leadership to empowerment was statistically significant (X→M, *B* = 0.30, *p* < .001), while the path from empowerment to organizational commitment was also statistically significant (M→Y_2_, *B* = 0.58, *p* < .001). Furthermore, the mediating model centered on ethical leadership, empowerment, and organizational commitment revealed a significant positive total effect (*B* = 0.69, *p* < .001). In the bootstrapping results for an indirect effect (X→M→Y_2_), the path was statistically significant (95% CI [0.082, 0.275]). These results indicate that the relationship between ethical leadership and organizational commitment is positively mediated by empowerment.

**Table 4:** Mediating Effect of Empowerment on the Relationship Between Ethical Leadership and Clinical Nurse Organizational Commitment (*N* = 220)

Variable	Path	Empowerment (M)				Path	Organizational Commitment (Y_2_)			
		*B*	*SE*	β	*p*		*B*	*SE*	β	*p*
Ethical leadership (X)	a	0.30	0.05	0.39	<.001	c’	0.51	0.09	0.34	<.001
Empowerment (M)						b	0.58	0.12	0.30	<.001

Dependent Variable	Independent Variable	Mediator Variable	Indirect Effect							
			*B*		Boot *SE*		Boot 95% CI			
Organizational Commitment (Y2)	Ethical Leadership	Empowerment	0.17		0.05			[0.082, 0.275]		

*Note.* Tolerance = .59–.96; VIF = 1.04–1.69; Durbin-Watson = 1.906 (du = 1.816 < d < 4-du = 2.184); *B* = unstandardized estimates; *SE* = standardized error; β = standardized estimates; CI = confidence interval; X = independent variable; M = mediator variable; Y_2_ = second dependent variable; Covariate variables = total time working in the current unit ([≥ 5] = 0, [1 to < 3] = 1, [3 to < 5] = 2), nursing management fee (2nd or 3rd grade = 0, 1st grade = 1).

## Discussion

The objective of this study was to clarify the relationship between the ethical leadership of nursing managers and the key outcomes (as described in leadership theory and previous studies on clinical nurses) of professional performance and organizational commitment in clinical nurses. Furthermore, this study focused on empowerment as a mediator of the association between ethical leadership and these outcomes.

The findings suggest that the positive effects of ethical leadership on nursing performance and organizational commitment are mediated by empowerment. This emphasizes the importance of ethical leadership in nursing management as well as the role of empowerment. Notably, even though the direct relationship between ethical leadership and nursing performance was not statistically significant, the indirect effect of empowerment was evident. These findings represent new insights in leadership research.

Nurse performance is a rarely addressed topic in other countries. In addition, organizational commitment may vary based on country-specific nursing work environments. Therefore, further studies are required to investigate and compare professional performance and organizational commitment in clinical nurses in different nursing settings around the world. Analysis of the correlations between the main variables revealed a significant correlation between ethical leadership in nursing managers and all three variables. The results showed that higher ethical leadership perceived by clinical nurses to be associated with higher nursing management and higher organizational commitment, which is consistent with previous studies (Amoah et al., 2022; Mostafa et al., 2021). The strongest correlation was found between empowerment and professional performance, implying that empowerment is a key factor in enhancing professional performance in clinical nurses, which is also consistent with prior studies (Al-Hammouri et al., 2021; Song et al., 2023; Wong & Laschinger, 2013).

The results of the mediation analysis verified that empowerment mediated the influence of ethical leadership on professional performance and organizational commitment in clinical nurses. The independent effect of ethical leadership on clinical nurse empowerment has not been previously reported. This study thus provides novel empirical evidence in support of the positive effect of empowerment in the field of nursing. Further, the significant effect of empowerment on nurse performance is consistent with the results of Song et al. (2023).

Detailed discussions of each major finding are provided as follows. First, in the hypothesized model of nursing performance, clinical nurse empowerment was found to mediate the influence of ethical leadership on clinical nursing performance. Similarly, a previous study on nurses working in acute care hospitals in Canada found empowerment to have a mediating effect on the relationship between head nurses’ authentic leadership and staff nurses’ nursing performance (Wong & Laschinger, 2013). In a meta-analytic path analysis conducted in South Korea, the indirect path from transformative leadership to nursing performance through empowerment was shown to be statistically significant, consistent with the results of this study (Kim & Jeong, 2020). These findings collectively suggest that empowerment plays a key role in mediating the influence of ethical leadership on the professional performance of clinical nurses. Thus, as part of training to improve nursing outcomes, education on empowerment should be reinforced to enhance the ability of managers to empower nurses.

Second, in the hypothesized model of organizational commitment, empowerment was found to mediate the relationship between ethical leadership in nursing managers and organizational commitment in clinical nurses. This finding is consistent with Lee et al. (2015), which found empowerment to partially mediate the effect of head nurses’ servant leadership on staff nurse organizational commitment. Similarly, Choi and Ahn (2016)’s study of nurses in general hospitals in the Seoul metropolitan area identified the mediating role of empowerment in the relationship between head nurses’ authentic leadership and staff nurses’ organizational commitment. However, the results of this study diverge from those of the meta-analytic path analysis, which found the indirect path from transformative leadership to organizational commitment through empowerment to fall short of statistical significance (Kim & Jeong, 2020). These results suggest the mediating influence of empowerment may vary based on leadership style. Thus, further studies are needed to explore the effect of empowerment on the relationship between various leadership styles and organizational commitment. The findings of this study collectively suggest that empowerment plays a key role as a mediator, together with ethical leadership, in enhancing the performance and organizational commitment of clinical nurses. Thus, cooperation between clinical nurses and nursing unit managers to further empower clinical nurses and strengthen their connections to their organization is recommended to enhance the performance of clinical nurses and their commitment to their nursing units, nursing organizations, and hospitals. Future studies should identify other variables that mediate the relationship between ethical leadership in nursing managers and clinical nurse performance and organizational commitment.

### Conclusions

This study contributes significantly to the field of organizational science by exploring the mediating role of empowerment in the relationship between ethical leadership in nursing managers and the professional performance and organizational commitment of clinical nurses. The findings reveal ethical leadership to have a positive direct and indirect influence on nursing performance and organizational commitment, thus underlining the imperative of empowering clinical nurses. This empowerment process is crucial to enhancing professional performance and fostering deeper organizational commitment, with ethical leadership serving as a pivotal element. To our knowledge, this research is unique in demonstrating the mediating role of empowerment in the context of clinical nursing.

Although this study draws from a comprehensive nationwide sample of clinical nurses across South Korea, which enhances its external validity, limitations in representativeness should be recognized. In future clinical practice, targeted educational programs designed to strengthen ethical leadership among nursing unit managers should be implemented to improve nursing performance and enhance organizational commitment. Moreover, the development and application of empowerment programs will be crucial to nurturing a sense of agency and effectiveness in clinical nurses.

In terms of future research, exploring additional mediators and moderators beyond empowerment in the nexus between ethical leadership in nursing managers and the outcomes of nursing performance and organizational commitment is essential. Also, expanding future research to include diverse health care settings and cultural contexts will be necessary to further validate and broaden scholarly understanding of these relationships.

This study underscores the vital roles of both leadership and empowerment in enhancing nursing performance and commitment in nursing practice settings, deepening our understanding of these dynamics in the health care sector. In delineating these relationships, this research sets a new benchmark for future investigations and practical applications in the realm of nursing and health care management.
